# The development of an effective synthetic route of lesinurad (RDEA594)

**DOI:** 10.1186/s13065-017-0316-y

**Published:** 2017-09-05

**Authors:** Qing Meng, Tong Zhao, Dongwei Kang, Boshi Huang, Peng Zhan, Xinyong Liu

**Affiliations:** Department of Medicinal Chemistry, Key Laboratory of Chemical Biology (Ministry of Education), School of Pharmaceutical Sciences, Shandong University, No. 44 West Culture Road, Jinan, 250012 Shandong People’s Republic of China

**Keywords:** Lesinurad, Uric acid salt transport protein 1, Gout, Synthesis

## Abstract

**Background:**

Lesinurad is a novel selective uric acid salt transport protein 1 (URAT1) inhibitor which is approved in the USA for the treatment of gout. However, there are some shortcomings among the reported synthetic routes, such as expensive materials, environmental pollution and poor yield.

**Results:**

In this study, an efficient, practical and environmentally-friendly synthetic route of lesinurad is reported. The main advantages of this route include inexpensive starting materials, mild conditions and acceptable overall yield (38.8%).

**Conclusion:**

Generally, this procedure is reasonable, reliable and suitable for industrial production.

**Graphical abstract:**
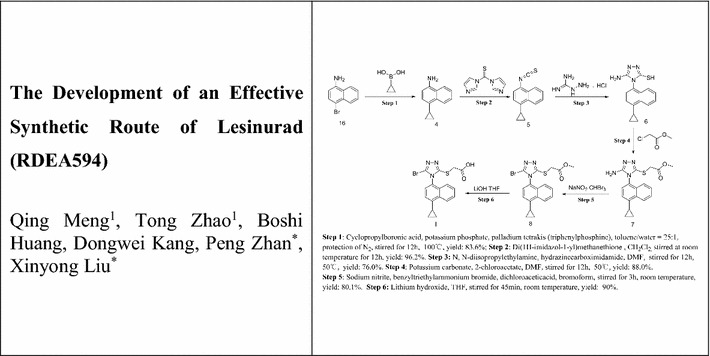
The improved synthetic procedure of lesinurad (I).

**Electronic supplementary material:**

The online version of this article (doi:10.1186/s13065-017-0316-y) contains supplementary material, which is available to authorized users.

## Background

Gout is a worldwide severe disease and affects millions of people especially in adult men. It is a crystal correlation arthropathy resulting from crystallization and deposition of monosodium urate (MSU), and is related to the purine metabolic disorder and the reduction of uric acid excretion. Sustained hyperuricemia is the most important biochemical basis of gout: normal adults produce about 750 mg of uric acid every day, of which approximately two-thirds of total urate is endogenous, while the remaining is from dietary purines. Irregular metabolism and decomposition can destroy the stability of uric acid level in the body and therefore result in hyperuricemia and gout. The population of gout patients has been rapidly increasing over the decades, while the existing drugs are limited. In this way, new treatment for hyperuricemia and gout is imperative [1–6].

**Fig. 1:**
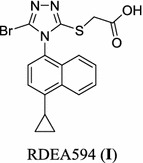
Structure of lesinurad (**I**)

**Scheme 1:**
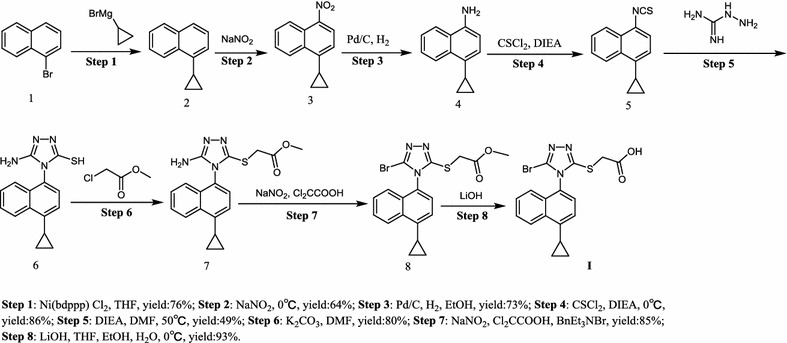
Synthesis of lesinurad (**I**) using 1-bromonaphthalene (**1**) as starting material [16–18]

**Scheme 2:**
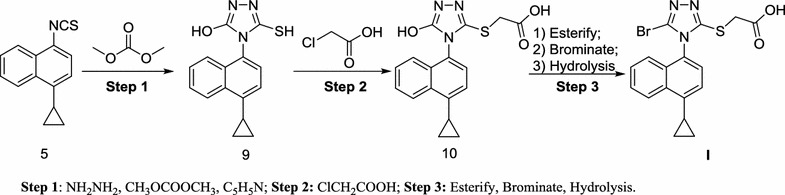
Synthesis of lesinurad (**I**) with **5** as starting material [19]

**Scheme 3:**
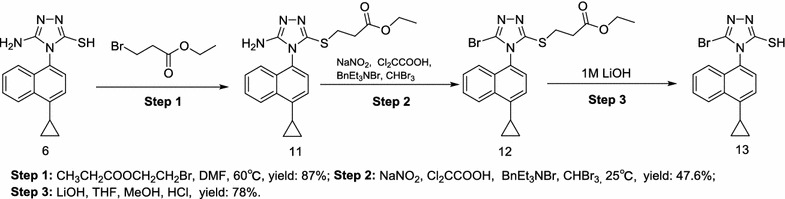
Synthesis of lesinurad (**I**) with **6** as starting material [16]

**Scheme 4:**
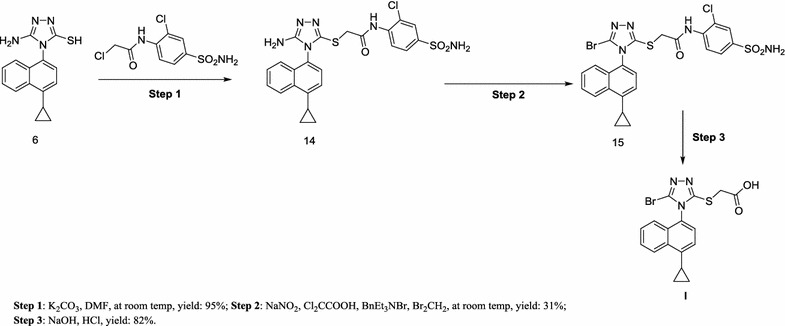
Alternative synthesis of lesinurad (**I**) with **6** as intermediate [20]

Lesinurad (RDEA594), 2-((5-bromo-4-(4-cyclopropylnaphthalen-1-yl)-4*H*-1,2,4-triazol-3-yl)thio)acetic acid, a first-in-class uric acid salt transport protein 1 (URAT1) inhibitor with potency of increasing the excretion of uric acid, has been approved by the US FDA in 2015 (Fig. 1) [7–13]. Lesinurad proved to be effective to block the reabsorption process along the nephron. Several research-scale synthetic methods have been reported for the preparation of Lesinurad (Schemes 1, 2, 3 and 4), which were associated with several drawbacks, such as the expensive materials, usage of hazardous reagents and poor yields [14, 15]. Therefore, there was a strong demand for the development of a more cost-effective and less toxic alternative production process for lesinurad with higher overall yield.

## Medicinal chemistry synthesis of lesinurad

The main medicinal chemistry routes of lesinurad is outlined in Schemes 1, 2, 3 and 4, which are mainly divided into three methods: (1) Method 1: the 1-bromonaphthalene (**1**) was used as the starting material (Scheme 1) [16–18]; (2) Method 2: 1-cyclopropyl-4-isothiocyanatonaphthalene (**5**) was employed as starting material (Scheme 2) [19]; (3) Method 3: 5-amino-4-(4-cyclopropylnaphthalen-1-yl)-4*H*-1,2,4-triazole-3-thiol (**6**) was used as the raw material or intermediate (Schemes 3, 4) [16, 20].

In Scheme 1, some limitations rendered this synthetic route unsuitable for larger-scale deliveries. (1) The low overall yield over the eight steps (just 9.5%) was not viable for a long-term synthesis; (2) in the first step, the reaction requires relatively harsh conditions (anhydrous oxygen free condition) and higher requirement for equipment; (3) the expensive starting material and the catalyst [1,3-bis(diphenylphosphino)propane]nickel(II) chloride were introduced at an early stage of the synthesis; (4) what is more, the use of extremely toxic, cacodorous and non-environmental-friendly reagent thiophosgene is highly undesirable for large-scale industrialization [16–18].

In another synthetic route (Scheme 2), the synthesis work started with the commercially available **5**. After esterification, bromination and hydrolysis, lesinurad (**I**) was finally obtained. Compared with the synthesis route in Scheme 1, there are no obvious advantages for this one [19].

In Schemes 3 and 4, the starting material **6** was treated in two different ways [16, 20]. However, both two routes are not practically valuable because of the commercially unavailable starting material, the long reaction time and low overall yield [20].

Therefore, these drawbacks prompted us to consider some alternative approaches to synthesize lesinurad and its intermediates. Herein, we present our efforts for the development of an efficient synthetic route with increased overall yield and reasonable reaction time. Results related to this work are summarized in this manuscript.

## Results and discussion

**Scheme 5:**
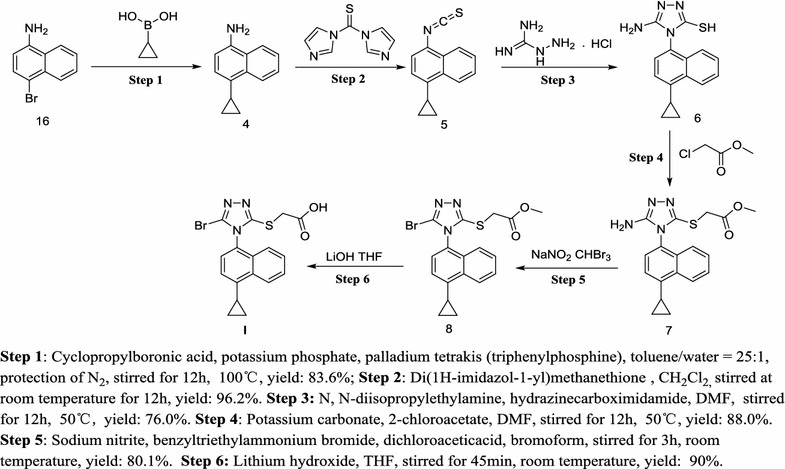
The improved synthetic procedure of lesinurad (**I**)

A novel synthetic procedure was successfully demonstrated to generate laboratory-scale lesinurad in six steps and a 38.8% overall yield, without using extremely poisonous organic reagents (Scheme 5). The route started with the cheaper and commercially available 4-bromonaphthalen-1-amine, which was first converted via Suzuki reaction in a mixed solvent (toluene/water = 25:1) to afford 4-cyclopropylnaphthalen-1-amine (**4**). Compound **4** reacted with di(1*H*-imidazol-1-yl)methanethione to obtain the key intermediate 1-cyclopropyl-4-isothiocyanatonaphthalene (**5**). Then treatment of **5** with hydrazinecarboximidamide afforded the intermediate 5-amino-4-(4-cyclopropylnaphthalen-1-yl)-4*H*-1,2,4-triazole-3-thiol (**6**). **6** successively underwent substitution and bromination reaction to give the intermediate methyl 2-((5-amino-4-(4-cyclopropylnaphthalen-1-yl)-4*H*-1,2,4-triazol-3-yl)thio)acetate (**7**) and brominated product **8**. At last, **8** was hydrolyzed to provide lesinurad (**I**). The total yield of the new route (up to 38.8%) was much better than those of the previously reported routes.

Comparing with the synthetic route in Scheme 1, we use 4-bromonaphthalen-1-amine as the starting material instead of unstable reagents such as cyclopropylmagnesium. Moreover, the route procedures are greatly shortened and improved. 1-Cyclopropyl-4-isothiocyanatonaphthalene (**5**) is an essential intermediate in the synthetic route of lesinurad (**I**), while thiophosgene was utilized to afford the key intermediate **5** in the previously reported synthetic routes. As is well known, thiophosgene is a reagent with low boiling point, volatility, smelly odor and strong toxicity. It is difficult to maintain a fully closed atmosphere during industrial production, and in this step, the actual amount of thiophosgene should be up to 2–3 times more than the theoretical amount, resulting in serious environmental pollution. In addition, when thiophosgene was employed to obtain the key intermediate **5**, some by-products also emerged, such as thiourea and its derivatives, which brought difficulties for separation and purification [21].

To begin with, 1,1′-thiocarbonyldiimidazole (TCDI) was selected as an alternative reagent to replace thiophosgene. The effects of different temperature (microwave, or not), reaction solvents (DMF, 1,4-dioxane, THF and dichloromethane) on the yields of product were analyzed. The results were depicted in Table 1.

**Table 1:** Optimization of reaction conditions

Entry	Solvent	Temperature (°C)	Time	Yield %
1	DCM	25	15 h	81.7
2	DCM	25	12 h	83.6
3	DCM	25	6 h	60.4
4	THF	25	12 h	78.1
5	THF	120	12 h	70.9
6	DMF	25	12 h	78.2
7	DMF	120	12 h	50.4
8	DMF	120 (microwave)	30 min	0^a^

^a^This reaction condition did not work

The common solvent DCM with lower boiling point was firstly applied at room temperature (25 °C) (entry 1–3). Obviously, the high yielding reaction time was 12 h (83.6%). Then THF and DMF with higher boiling point were utilized as solvents to perform this reaction under room temperature and 120 °C, respectively (entry 4–7). Compared with DCM, the yield was not increased in THF and DMF at room temperature. Higher temperature seemed to be detrimental to the yield. Unfortunately, the use of microwave radiation instrument in relatively short time and higher temperature didn’t have a beneficial effect on this reaction.

Then, we discuss the proper ratio between 4-cyclopropylnaphthalen-1-amine and TCDI (Table 2). We change the amount of TCDI to find the best scale. Obviously, the high yielding reaction ratio was 4-cyclopropylnaphthalen-1-amine/TCDI = 1:1.5. All in all, the optimum (high yielding) conditions for this study are as follows: the temperature of reaction is about 25 °C, the proper reaction time is 12 h, the solvent is DCM and the suitable ratio between 4-cyclopropylnaphthalen-1-amine and TCDI is 1:1.5.

**Table 2:** Optimization of reaction ratio

Entry	4-Cyclopropylnaphthalen-1-amine(eq.)	TCDI (eq.)	Yield %
1	1	1	72.9
2	1	1.5	83.6
3	1	2	83.5
4	1	2.5	82.7

## Conclusions

In conclusion, we provide an alternative method for the preparation of lesinurad, a newly-launched medicine for the treatment of gout. The method proceeds in six linear steps on gram scale with multiple advantages, including higher total yield of 38.8% (much better than those of the original ones). The most significant step of the route is the synthesis of key intermediate 1-cyclopropyl-4-isothiocyanatonaphthalene (**5**), and the main advantages of the method are readily available inexpensive starting materials, less toxic condition and high yield. Importantly, the reaction reactant, solvent, reaction time and temperature of this step were preliminarily investigated. This efficient and environmental-friendly process and the optimum conditions for the preparation of lesinurad may form the basis of a future manufacturing route. Further work in our lab would be required to remove the requirement for a silica treatment and then to perform a scale-up campaign (Additional file 1).

## Experimental section

All melting points (mp) were determined on a micromelting point apparatus and are uncorrected. Mass spectra were performed on a LC Autosampler Device: Standard G1313A instrument by electrospray ionization. ^1^H NMR and ^13^C NMR spectra were obtained on a Bruker AV-400 spectrometer (Bruker BioSpin, Fällanden, Switzerland) in the indicated solvent DMSO-*d*_6_. Chemical shifts were expressed in *δ* units (ppm), using TMS as an internal standard, and *J* values were reported in hertz (Hz). TLC was performed on Silica Gel GF254. Spots were visualized by irradiation with UV light (λ 254 nm). Flash column chromatography was carried out on columns packed with silica gel 60 (200–300 mesh). The microwave reaction was conducted on a CEM Discover (0–600 W, 2450 MHz) instrument and the conventional high pressure reaction was performed on Parr 4590 instrument. Solvents were of reagent grade and, if needed, were purified and dried by distillation. Starting materials, solvents, and the key reagents were purchased from commercial suppliers and were used as received without purification. Rotary evaporators were served in concentration of the reaction solutions under reduced pressure.

### 4-Cyclopropylnaphthalen-1-amine (**4**)

4-Bromonaphthalen-1-amine (16) (90 mmol, 20.0 g), cyclopropylboronic acid (116 mmol, 10.0 g), potassium phosphate (300 mmol, 64.0 g) and palladium-tetrakis(triphenylphosphine) (6 mmol, 7.0 g) were dissolved in 104 mL mixed solvent (toluene/water = 25:1) under the protection of N_2_. The reaction was heated at 100 °C for 12 h. Subsequently, the solution was filtered and concentrated under reduced pressure. Then, water (100 mL) was added and the solution was extracted using EtOAc (3 × 30 mL), washed with saturated brine (50 mL), dried over anhydrous Na_2_SO_4_, filtered and concentrated under reduced pressure to obtain the crude product (13.8 g), which was purified by flash column chromatography (EA:PE = 1:4) to afford **4** as the clear brown oil. Yield: 83.6%. ^1^H NMR (400 MHz, DMSO) *δ* 8.25 (d, *J* = 7.9 Hz, 1H, Naph-H), 8.07 (d, *J* = 8.2 Hz, 1H, Naph-H), 7.49 (ddd, *J* = 8.2, 6.8, 1.1 Hz, 1H, Naph-H), 7.39 (ddd, *J* = 8.1, 6.8, 1.2 Hz, 1H, Naph-H), 7.00 (d, *J* = 7.6 Hz, 1H, Naph-H), 6.59 (d, *J* = 7.7 Hz, 1H, Naph-H), 5.54 (s, 2H, NH_2_), 2.17–2.10 (m, 1H, CH), 0.94–0.90 (m, 2H, CH_2_), 0.57–0.53 (m, 2H, CH_2_). ^13^C NMR (100 MHz, DMSO) *δ* 143.73, 134.23, 126.15, 125.86, 125.22, 124.70, 123.96, 123.57, 123.23, 107.43, 13.03, 6.46 (2×C). ESI–MS: m/z 184.2 [M + H]^+^. C_13_H_13_N (Exact Mass: 183.10).

### 1-Cyclopropyl-4-isothiocyanatonaphthalene (**5**)

Di(1*H*-imidazol-1-yl)methanethione (50 mmol, 8.8 g) was added to a solution of **4** (33 mmol, 6.0 g) in dichloromethane (100 mL). The mixture was stirred at room temperature for 12 h. Subsequently, the solution was filtered and concentrated under reduced pressure. Then, the reaction was added with water (100 mL), and extracted with EtOAc (3 × 30 mL). The organic layers were combined, washed with saturated brine (50 mL), dried over with anhydrous Na_2_SO_4_, filtered and concentrated under reduced pressure. Finally, the residue was further purified by silica gel chromatography (EA:PE =1 :8) to afford **5** as a clear brown oil, (7.1 g). Yield: 96.2%. ^1^H NMR (400 MHz, DMSO) *δ* 8.48 (d, *J* = 9.4 Hz, 1H, Naph-H), 8.02 (d, *J* = 9.4 Hz, 1H, Naph-H), 7.76–7.71 (m, 2H, Naph-H), 7.55 (d, *J* = 7.7 Hz, 1H, Naph-H), 7.24 (d, *J* = 7.7 Hz, 1H, Naph-H), 2.46–2.39 (m, 1H, CH), 1.11–1.07 (m, 2H, CH_2_), 0.77–0.73 (m, 2H, CH_2_). ^13^C NMR (100 MHz, DMSO) *δ* 140.42, 133.72, 128.78, 128.08, 127.75, 126.33, 125.56, 124.76, 124.22, 123.27, 122.79, 13.28, 7.57 (2×C). C_14_H_11_NS (Exact Mass: 225.06).

### 5-Amino-4-(4-cyclopropylnaphthalen-1-yl)-4*H*-1,2,4-triazole-3-thiol (**6**)

To a suspension of *N*,*N*-diisopropylethylamine (39.9 mmol, 5.1 g) in anhydrous DMF (5 mL) was dropwise added a solution of compound **5** (13.3 mmol, 3.0 g) and hydrazinecarboximidamide (26.6 mmol, 2.9 g) in DMF (50 mL) at 50 °C. Additional DMF (5 mL) was used to rinse the flask and then was added to the solution. The resulting mixture was stirred for cc and after removing the solvent, 2 N NaOH (20 mL) was added for further reaction until its completion. Then the mixture was filtered and acidified to pH 4–5 with 2 N HCl to form precipitate and then filtered, dried at 45–50 °C under vacuum and recrystallized from ethyl alcohol (EtOH) to afford **6** as a white solid (2.85 g). Yield: 76.0%. ^1^H NMR (400 MHz, DMSO) *δ* 12.89 (s, 1H, SH), 8.52 (d, *J* = 8.4 Hz, 1H, Naph-H), 7.66 (t, *J* = 7.6 Hz, 1H, Naph-H), 7.58 (t, *J* = 7.6 Hz, 1H, Naph-H), 7.38 (s, 2H, Naph-H), 7.32 (d, *J* = 8.2 Hz, 1H, Naph-H), 5.88 (s, 2H, NH_2_), 2.54–2.47 (m, 1H, CH), 1.15–1.12 (m, 2H, CH_2_), 0.83–0.81 (m, 2H, CH_2_). ^13^C NMR (101 MHz, DMSO) *δ* 165.38, 153.12, 141.74, 134.26, 130.17, 128.22, 128.03, 127.35, 126.97, 125.26, 123.42, 123.33, 13.39, 7.59, 7.36. ESI–MS: m/z 283.4 [M+H]^+^. C_15_H_14_N_4_S (Exact Mass: 282.09).

### Methyl 2-((5-amino-4-(4-cyclopropylnaphthalen-1-yl)-4*H*-1,2,4-triazol-3-yl)thio)acetate (**7**)

A mixture of **6** (7.1 mmol, 2.0 g) and potassium carbonate (7.8 mmol, 1.1 g) was dissolved in 40 mL DMF, and the methyl 2-chloroacetate (7.4 mmol, 0.8 g) was added dropwise. Then the mixed solution was heated at 50 °C for 12 h. After reaction, the mixture was poured into 100 mL water to precipitate and then the formed solid was filtered, dried at 45–50 °C under vacuum and recrystallized from ethyl alcohol (EtOH) to afford **7** as a white solid (2.21 g). Yield: 88.0%. ^1^H NMR (400 MHz, DMSO) *δ* 8.55 (d, *J* = 8.4 Hz, 1H, Naph-H), 7.71 (t, *J* = 7.1 Hz, 1H, Naph-H), 7.63 (t, *J* = 7.6 Hz, 1H, Naph-H), 7.48 (d, *J* = 7.6 Hz, 1H, Naph-H), 7.40 (d, *J* = 7.6 Hz, 1H, Naph-H), 7.21 (d, *J* = 8.1 Hz, 1H, Naph-H), 5.78 (s, 2H, NH_2_), 3.84–3.69 (m, 2H, CH_2_), 3.58 (s, 3H, CH_3_), 2.59–2.51 (m, 1H, CH), 1.14 (dd, *J* = 8.4, 1.8 Hz, 2H, CH_2_), 0.88–0.79 (m, 2H, CH_2_). ^13^C NMR (100 MHz, DMSO) *δ* 169.18, 157.31, 143.38, 142.26, 134.21, 129.94, 127.86, 127.32 (2×C), 127.27, 125.40, 123.32, 122.80, 52.75, 34.78, 13.37, 7.57 (2×C). ESI–MS: m/z 355.5 [M+H]^+^. C_18_H_18_N_4_O_2_S (Exact Mass: 354.12).

### Methyl 2-((5-bromo-4-(4-cyclopropylnaphthalen-1-yl)-4*H*-1,2,4-triazol-3-yl)thio)acetate (**8**)

To a suspension of **7** (5.6 mmol, 2.0 g), sodium nitrite (112 mmol, 7.7 g), benzyltriethylammonium bromide (16.8 mmol, 4.5 g) in bromoform (30 mL) was dropwise added a solution of dichloroaceticacid (11.2 mmol, 1.4 g) at room temperature. Water (100 mL) was added and the solution was extracted using EtOAc (3 × 30 mL), washed with saturated brine (50 mL), dried over anhydrous Na_2_SO_4_, filtered and concentrated under reduced pressure to obtain the crude product which was purified by flash column chromatography (MeOH:CH_2_Cl_2_ = 1:50) and recrystallized from ethyl alcohol (EtOH) to afford the target compounds **8** (1.89 g). Yield: 80.1%. ^1^H NMR (400 MHz, DMSO) *δ* 8.59 (d, *J* = 8.4 Hz, 1H, Naph-H), 7.79–7.72 (m, 1H, Naph-H), 7.66 (dd, *J* = 14.0, 7.4 Hz, 2H, Naph-H), 7.44 (d, *J* = 7.6 Hz, 1H, Naph-H), 7.15 (d, *J* = 8.1 Hz, 1H, Naph-H), 4.07 (d, *J* = 3.9 Hz, 2H, CH_2_), 3.63 (s, 3H,CH_3_), 2.59–2.54 (m, 1H, CH), 1.15 (dd, *J* = 8.4, 2.0 Hz, 2H, CH_2_), 0.87 (d, *J* = 14.3 Hz, 2H, CH_2_). ^13^C NMR (100 MHz, DMSO) *δ* 168.77, 153.68, 143.69, 133.97, 132.05, 129.09, 128.63, 127.78, 127.32, 126.99, 125.66, 123.15, 122.18, 53.01, 34.05, 13.41, 7.83, 7.77. ESI–MS: m/z 418.5 [M+H]^+^. C_18_H_16_BrN_3_O_2_S (Exact Mass: 417.01).

### 2-((5-Bromo-4-(4-cyclopropylnaphthalen-1-yl)-4*H*-1,2,4-triazol-3-yl)thio)acetic acid Lesinurad (**I**)

Compound **8** (2.7 mmol, 1.14 g) was dissolved in THF (10 mL), then lithium hydroxide solution was added at 0 °C and the mixture was stirred at this temperature for 45 min. After removing the solvent, the residue was diluted with water (20 mL). Then the mixture was acidified to pH 2–3 with 2 N HCl to form precipitate and the formed solid was filtered, then recrystallized with ethyl acetate (EA) and dried at 55–60 °C under vacuum to give the target compound lesinurad (**I**). Yield: 90.0%. ^1^H NMR (400 MHz, DMSO) *δ* 8.58 (d, *J* = 8.4 Hz, 1H, Naph-H), 7.74 (t, *J* = 7.6 Hz, 1H, Naph-H), 7.67–7.63 (m, 2H, Naph-H), 7.44 (d, *J* = 7.6 Hz, 1H, Naph-H), 7.16 (d, *J* = 8.3 Hz, 1H, Naph-H), 3.98 (s, 2H, CH_2_), 2.59–2.53 (m, 1H, CH), 1.15 (dd, *J* = 8.4, 1.9 Hz, 2H, CH_2_), 0.89–0.85 (m, 2H, CH_2_). ^13^C NMR (100 MHz, DMSO) *δ* 169.44, 154.18, 143.61, 133.98, 131.76, 129.13, 128.58, 127.75, 127.30, 127.10, 125.64, 123.16, 122.24, 35.13, 13.41, 7.79, 7.77. ESI–MS: m/z 406.4 [M+H]^+^.C_17_H_14_BrN_3_O_2_S (Exact Mass: 403.00).

## Additional file

**Additional file 1.**  Copies of NMR and MS spectra.
