# Supplementary material for: The development of an effective synthetic route of lesinurad (RDEA594)
Source: Chem Cent J. 2017 Sep 5;11:86. doi: 10.1186/s13065-017-0316-y (PMC5583131; doi:10.1186/s13065-017-0316-y)
Supplement: Supplementary file 1 — Additional file 1. Copies of NMR and MS spectra. [file 13065_2017_316_MOESM1_ESM.docx]

Compound 4 ^1^HNMR


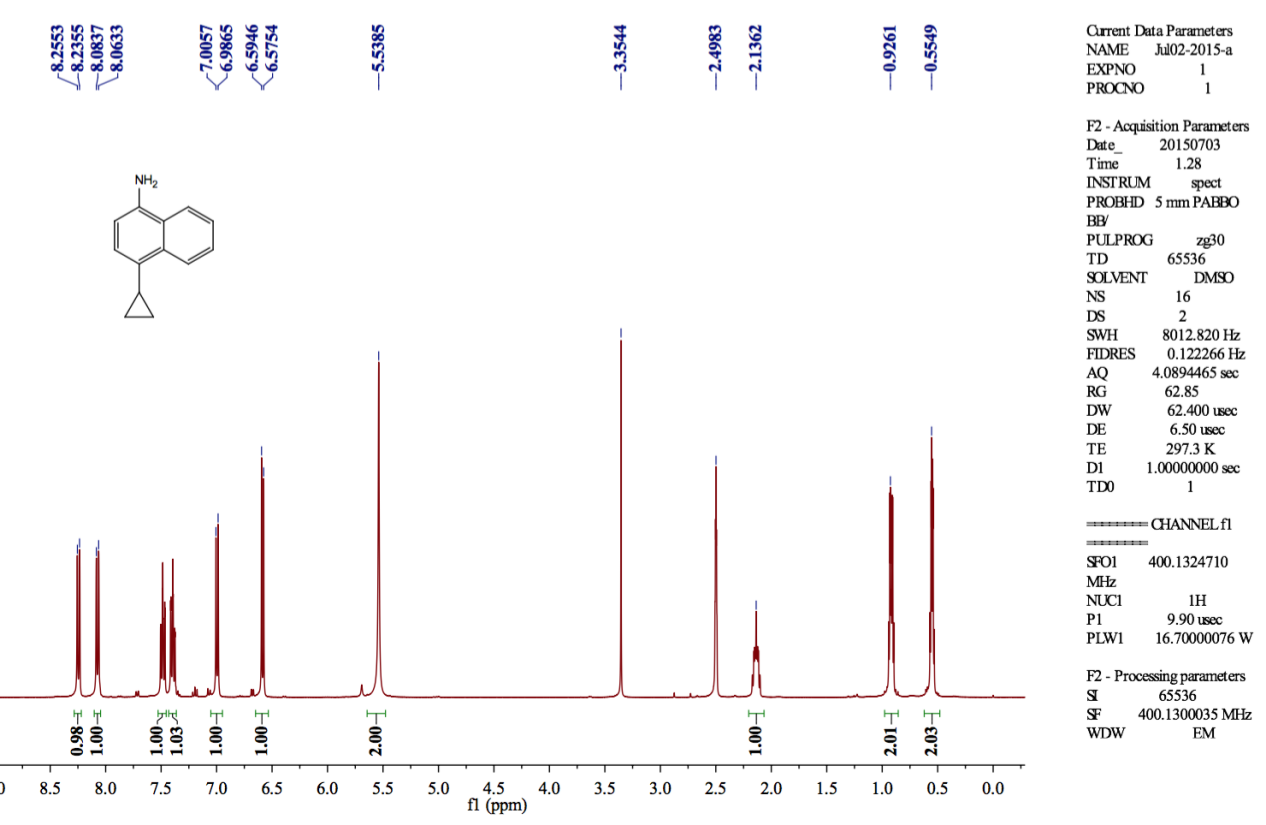


Compound 4 ^13^CNMR


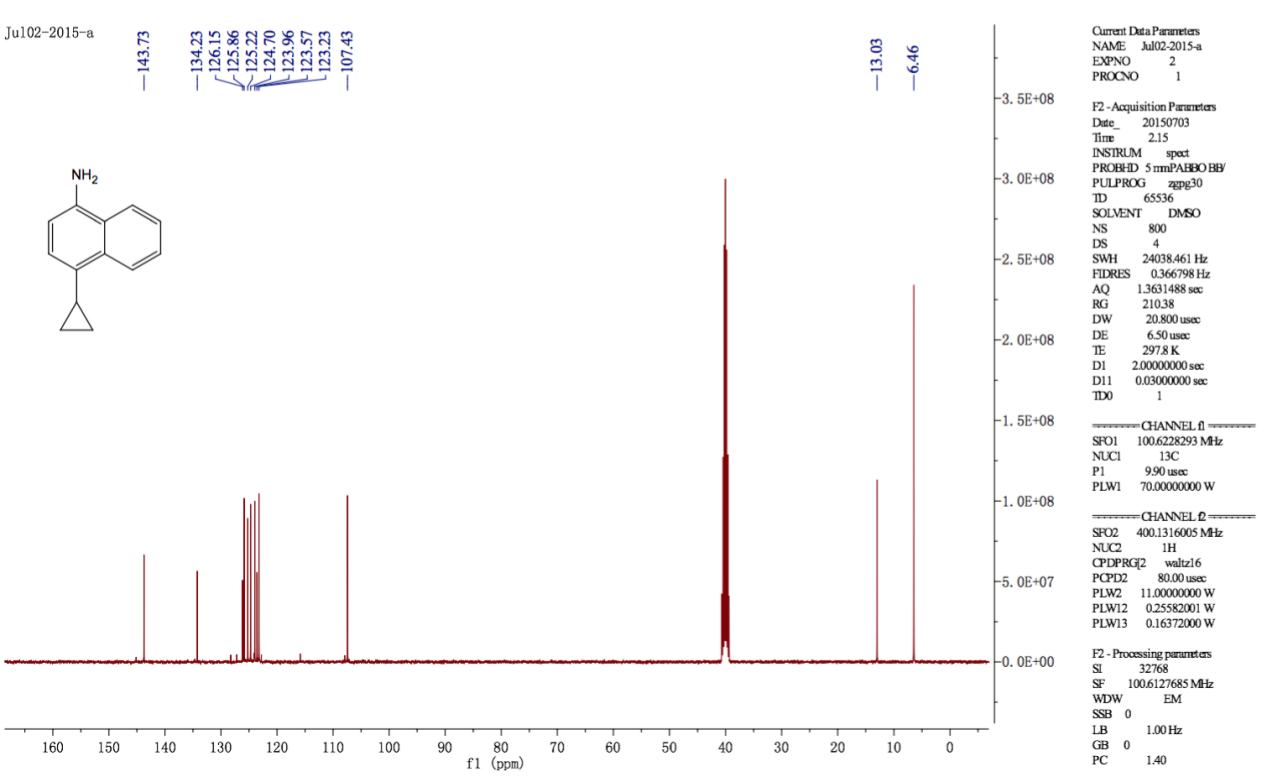


Compound 4 MS


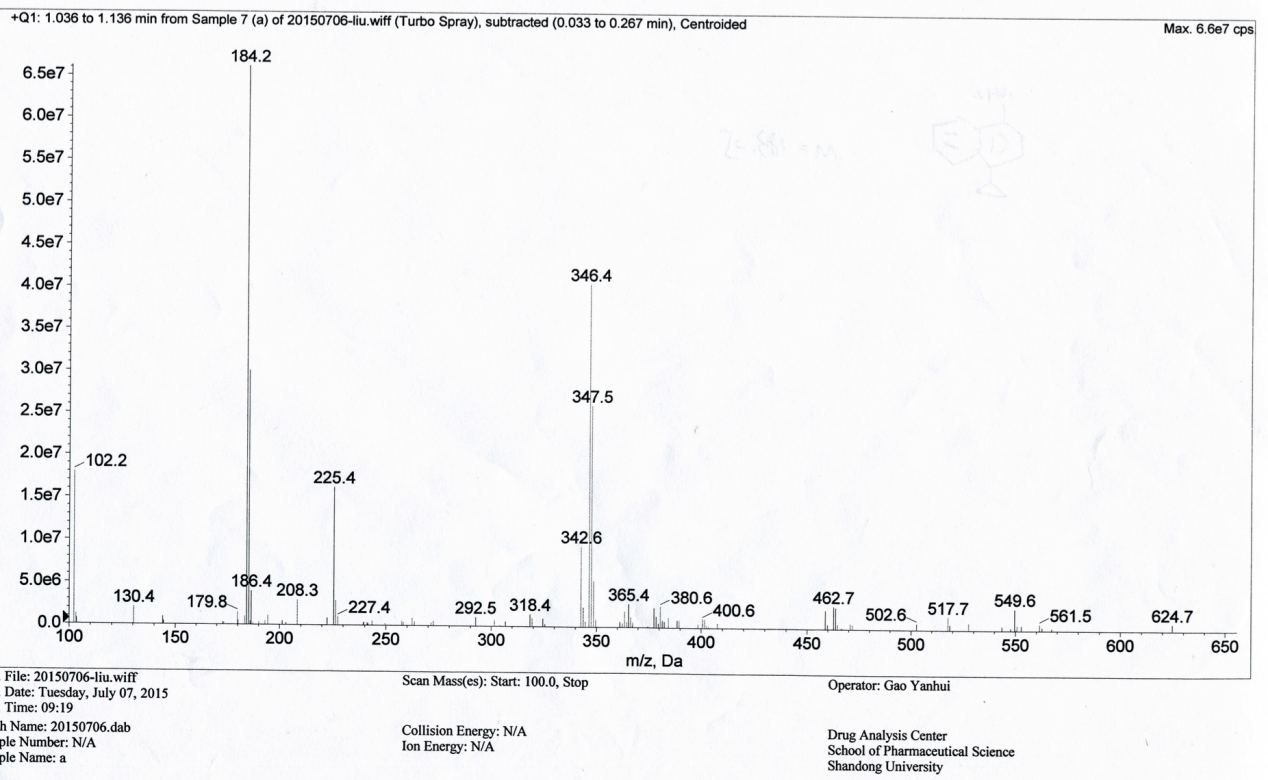


Compound 5 ^1^HNMR


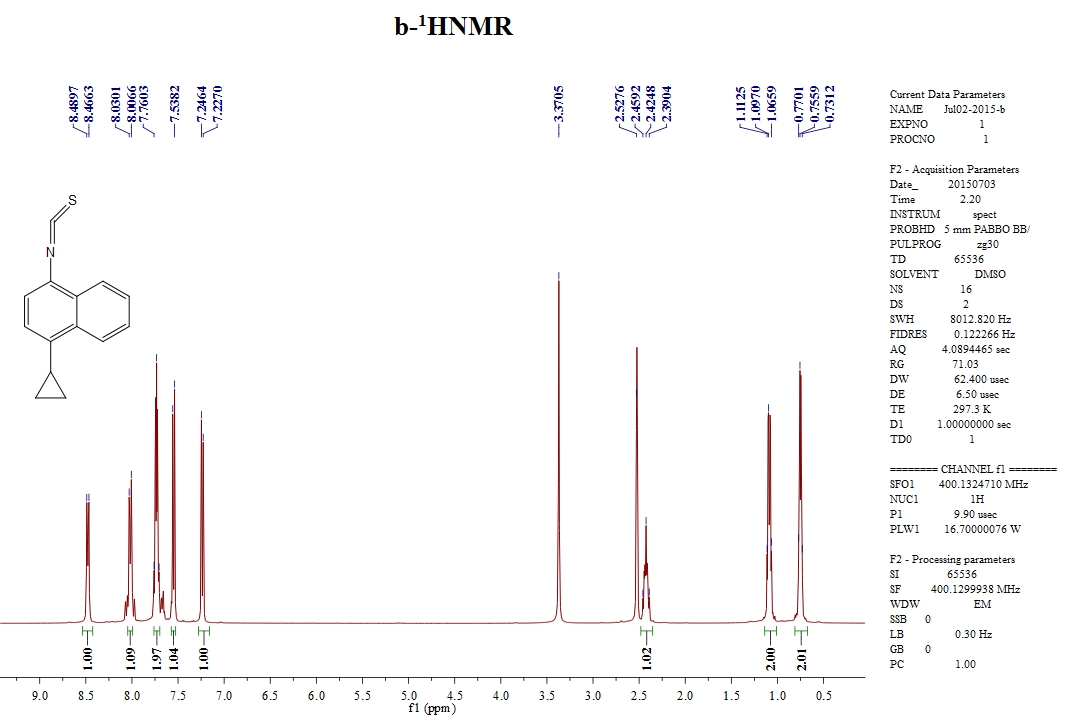


Compound 5 ^13^CNMR


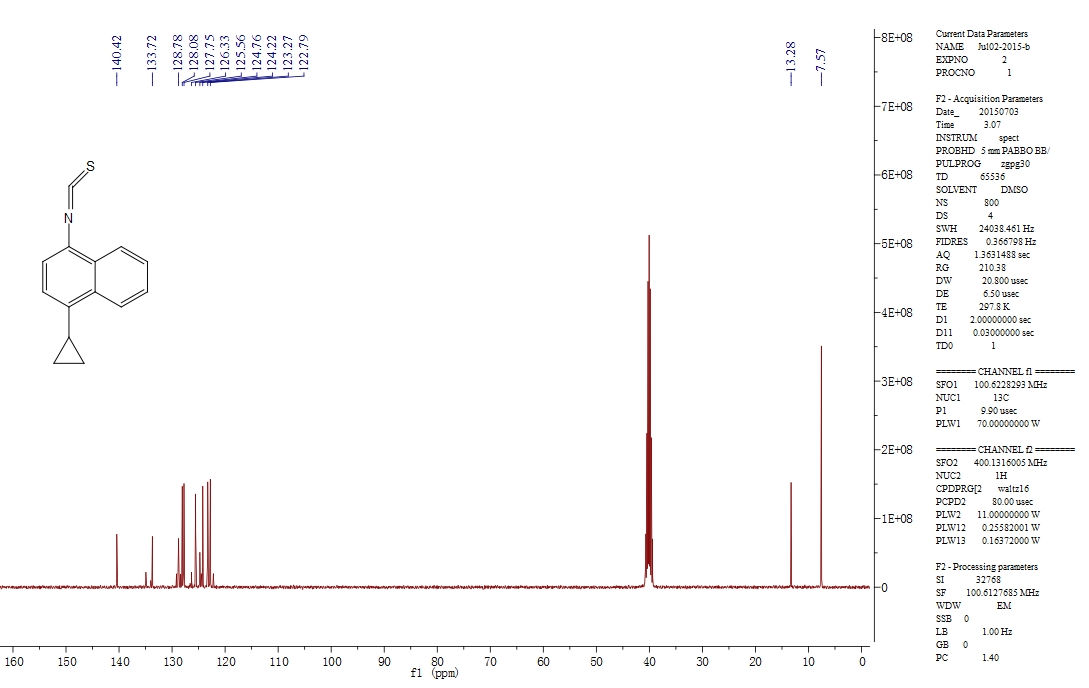


Compound 6 ^1^HNMR


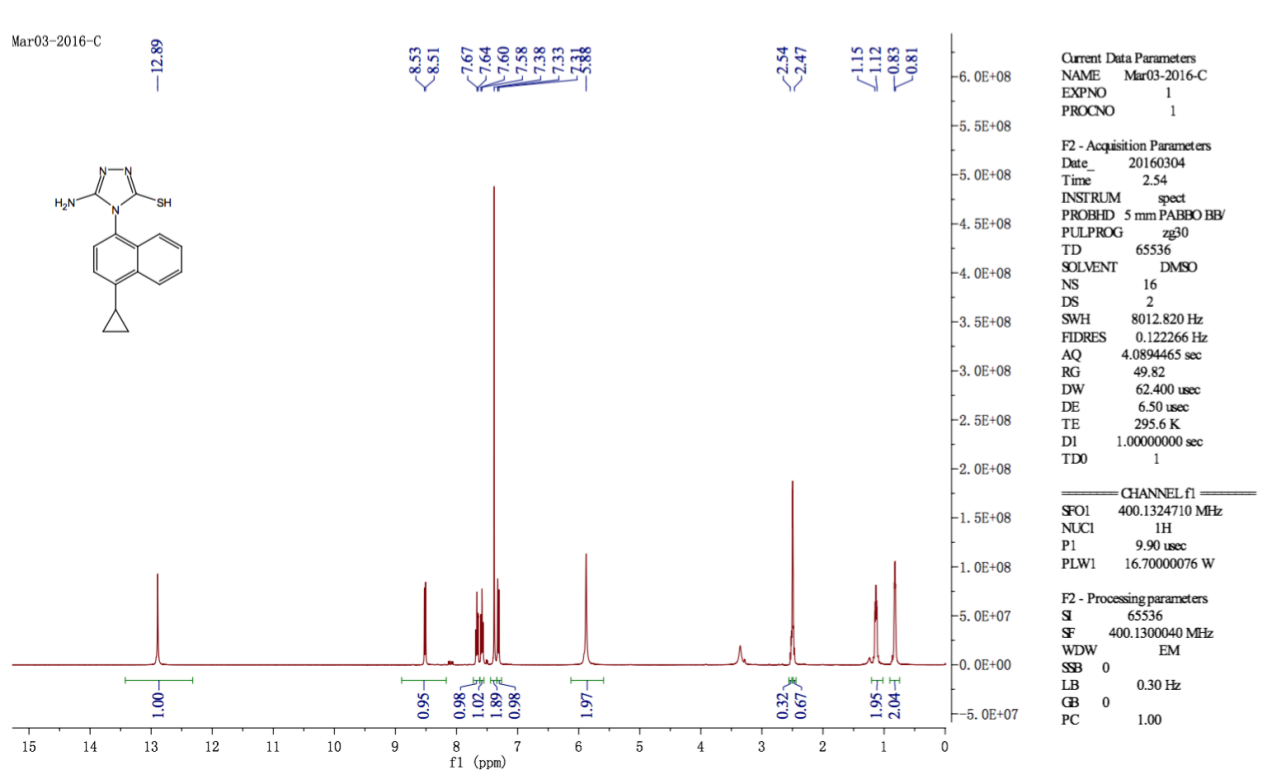


Compound 6 ^13^CNMR


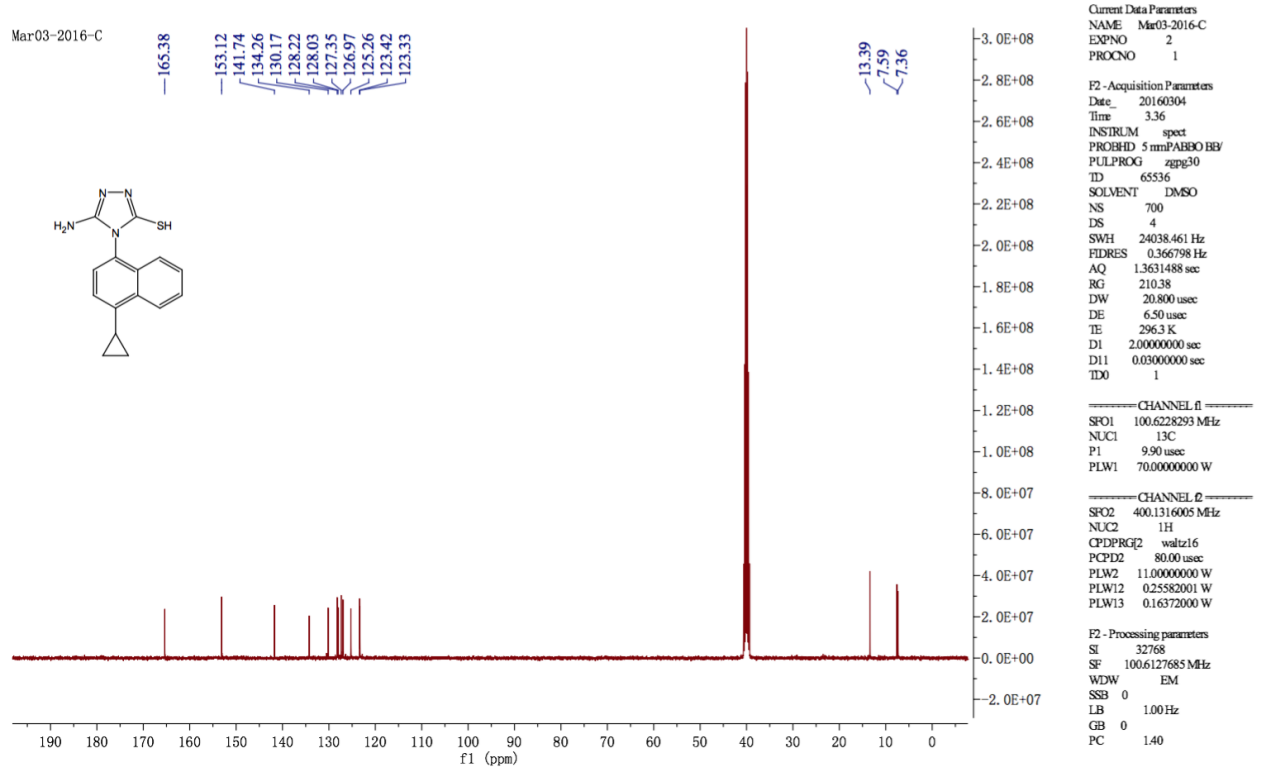


Compound 6 MS

Compound 7 ^1^HNMR


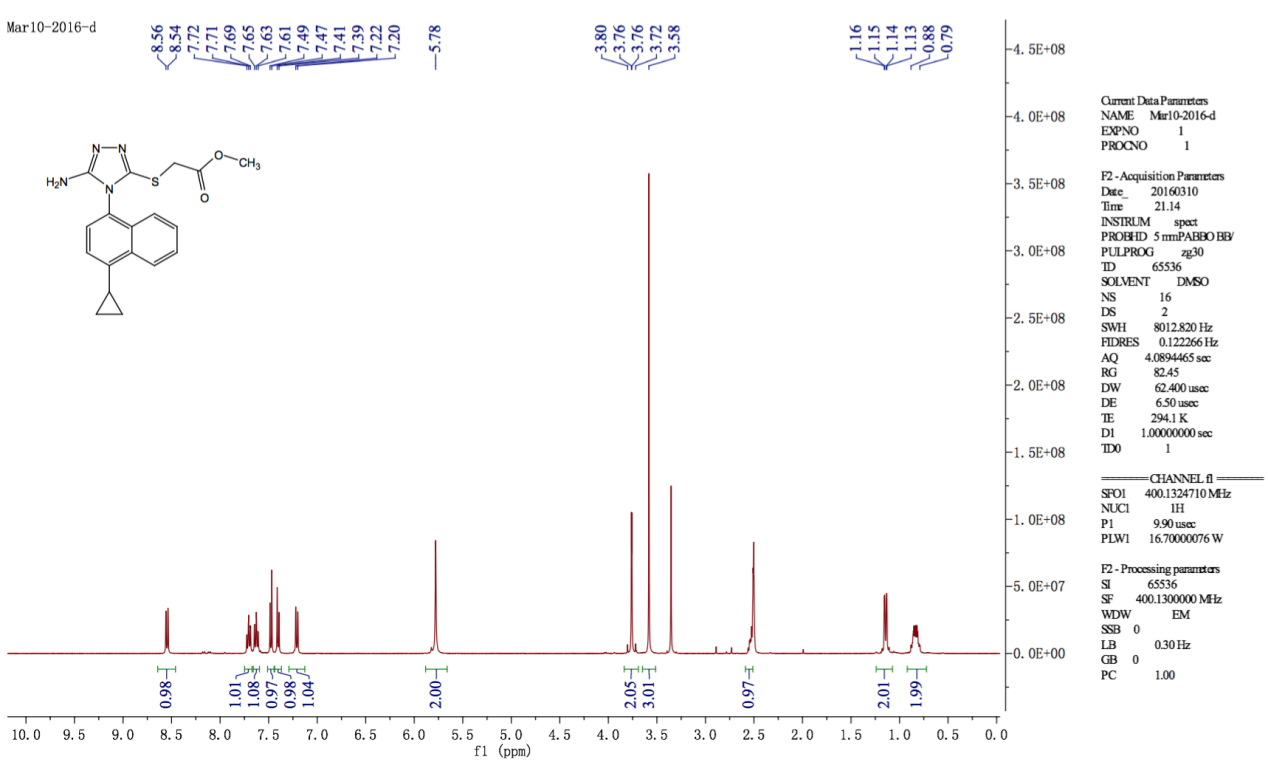


Compound 7 ^13^CNMR


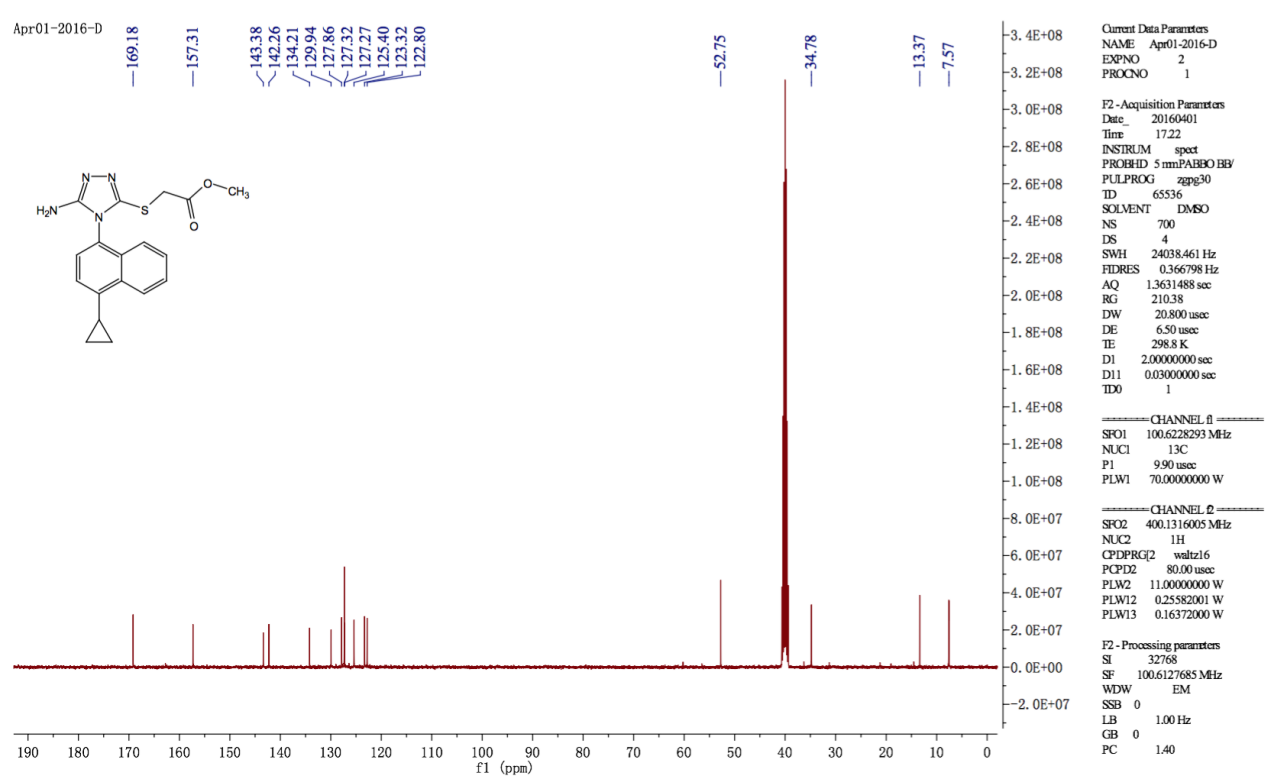


Compound 7 MS

Compound 8 ^1^HNMR


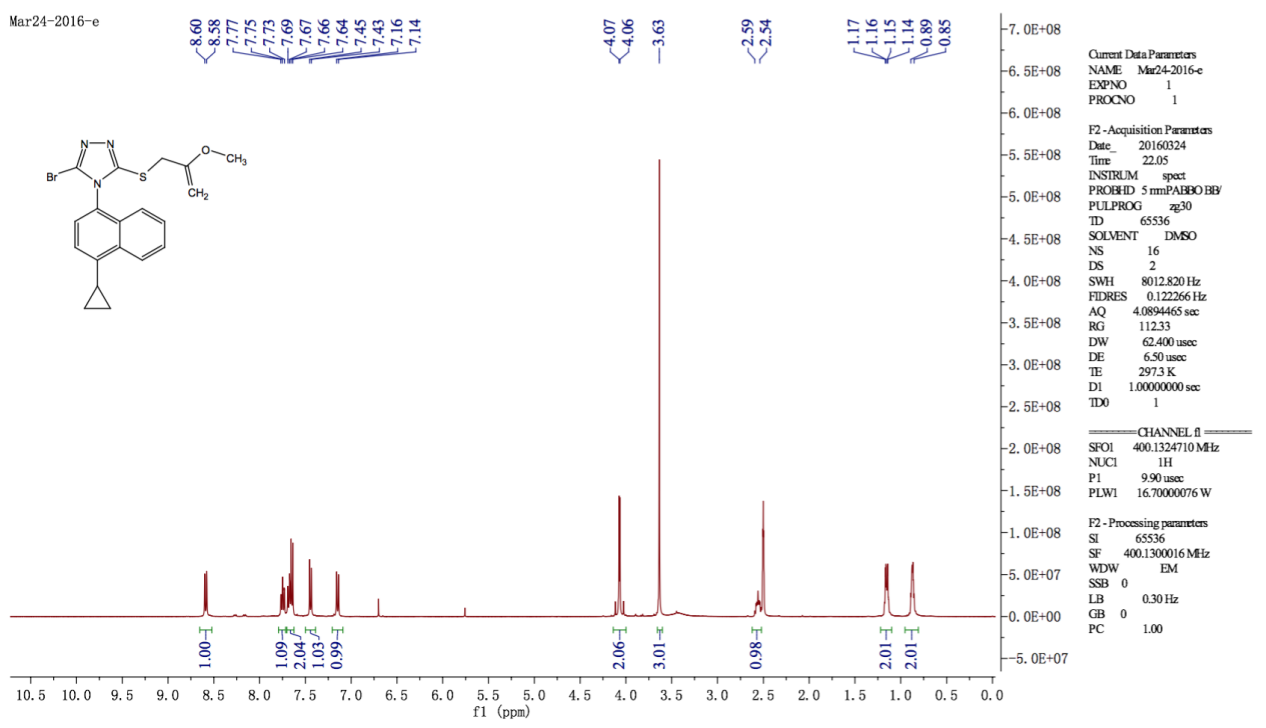


Compound 8 ^13^CNMR


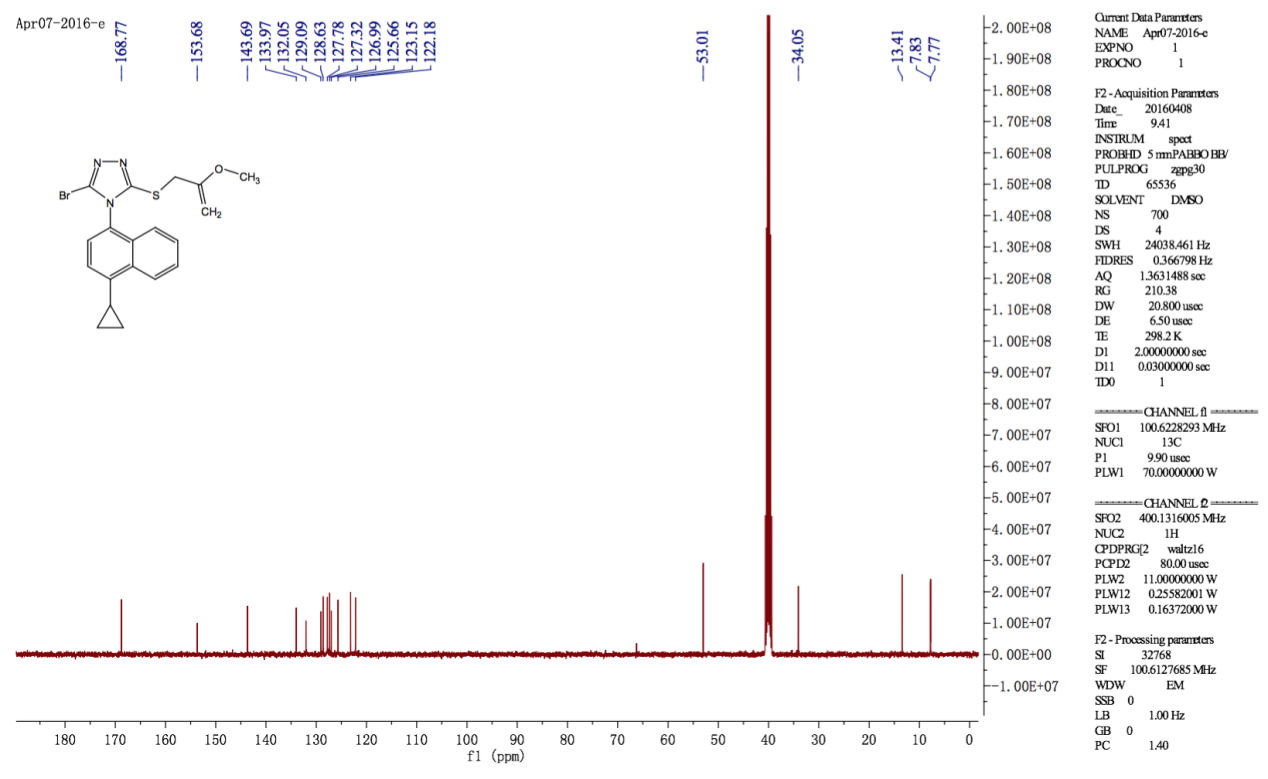


Compound 8 MS

Compound Ⅰ ^1^HNMR


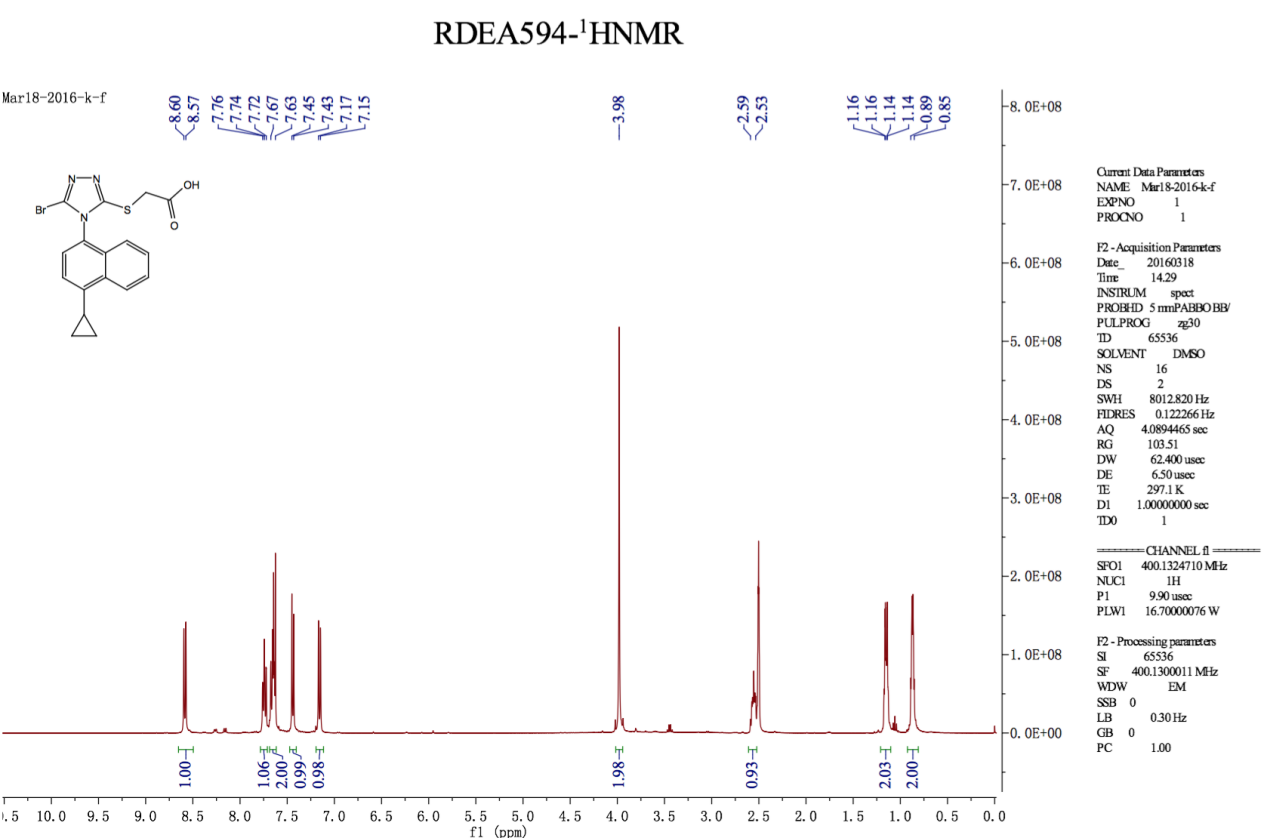


Compound Ⅰ ^13^CNMR


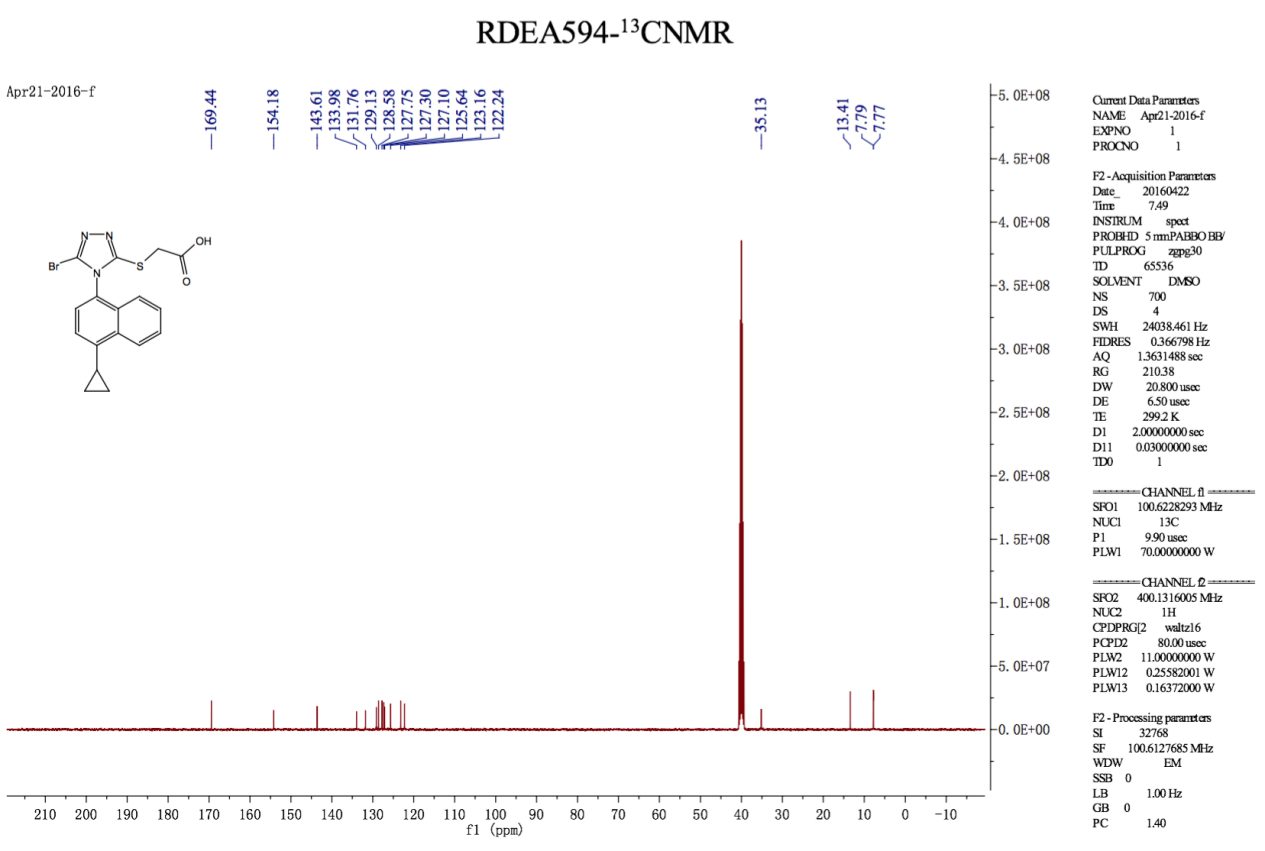


Compound Ⅰ MS
